# 32.2 ABNORMALITIES OF SYNAPTIC PROTEOMES IN SCHIZOPHRENIA

**DOI:** 10.1093/schbul/sby014.133

**Published:** 2018-04-01

**Authors:** Adam Funk, Kenneth Greis, Jarek Meller, Robert McCullumsmith

**Affiliations:** University of Cincinnati

## Abstract

**Background:**

The human brain is comprised of billions of neurons that form networks of connections within and between brain regions. These connections facilitate neuroplastic events that underlie learning and memory, critical aspects of cognitive function often perturbed in neuropsychiatric illnesses. Neuronal signaling is mediated by fast and slow transmission events, encompassing receptors, ligands, ions, enzymes, and other substrates. These elements are spatially arranged in subcellular microdomains, facilitating juxtaposition of proteins that coordinate various biological processes. For example, synaptic transmission is modulated via release of neurotransmitter into the synaptic cleft, where receptors are activated and the postsynaptic cell modulated via electrical and chemical signals. The pre- and postsynaptic compartments include highly specialized protein clusters, with elegant and complex regulatory mechanisms that traffic proteins to and from these zones. In particular, postsynaptic densities are microdomains comprised of about 1000 unique proteins that are interacting with one another via specialized multipotent scaffolding molecule. Postsynaptic density-95 (PSD-95) is a multipotent scaffolding, trafficking, and clustering protein that links glutamate receptors, signaling molecules, and other structural proteins at postsynaptic sites. More than 95% of PSD-95 expression is localized to excitatory synapses, and it is the most abundant scaffolding protein within the postsynaptic density. Mounting genetic, proteomic, and pharmacological evidence converges on alterations in the postsynaptic density of excitatory synapses in subjects with schizophrenia. Cognitive and negative symptoms associated with dysfunction of limbic circuitry, including working memory and motivation, are particularly implicated by this mechanism. To investigate excitatory postsynaptic protein hubs in schizophrenia, we assessed the PSD-95 protein interactome from brain tissue of subjects with schizophrenia and controls.

**Methods:**

Human brain tissue from fifteen subjects with schizophrenia and fifteen control subjects from the DLPFC was processed for affinity purification of PSD-95 protein complexes. We confirmed PSD-95 capture and enrichment from each sample using Western blot analyses. Samples were then pooled by region and assessed by mass spectrometry for a quality control step. Pooled samples were run in triplicate. Go versus nogo was based on finding more than 500 unique peptides in each pooled sample from each region. Next, individual samples were run through our mass spectrometry protocol in triplicate. We then subtracted any non-specifically captured peptides identified by our IgG control studies that were performed in parallel to PSD-95 affinity purification. Data were normalized within each of the mass spec runs to the most intense PSD-95 peptide. This PSD-95 peptide used for normalization was the same in every sample. Peptides that were present in at least 2 of 3 technical replicates were carried forward and subjected to quantile normalization. Peptides missing in a technical replicate were replaced by imputation, and the dataset subjected to unsupervised clustering. We also performed a semisupervised clustering protocol, non negative matrix factoring (NMF). Consensus signatures of 200 peptides were identified for each brain region, and subjected to traditional and alternative bioinformatics analyses to identify pathways, processes and compounds associated with the signatures for each brain region.

**Results:**

Preliminary analyses indicate changes in the PSD-95 interactome consistent with diminished NMDA receptor signaling complex activity in schizophrenia, with lower levels of NMDA-subtype glutamate receptor subunits, as well as protein kinases associated with postsynaptic signaling in this complex. Specific biological pathways and processes identified include metabolic and inflammatory pathways. We will also present proteomic signatures generated from this dataset, and interrogate the iLINCS perturbagen database to identify drugs and genes that simulate or reverse this signature.

**Discussion:**

Our preliminary data suggest that NMDA receptor function is compromised in schizophrenia. Additional work is needed to see if this is an effect of antipsychotic medications. Our proteomic findings extend the NMDA receptor hypothesis beyond the transcriptome, highlighting an important new approach for assessing abnormalities of synapses in postmortem brain.

